# Altered bodily perceptions in chronic neuropathic pain conditions and implications for treatment using immersive virtual reality

**DOI:** 10.3389/fnhum.2022.1024910

**Published:** 2022-11-17

**Authors:** Tony Donegan, Brenda E. Ryan, Maria V. Sanchez-Vives, Justyna Świdrak

**Affiliations:** ^1^Institut d’Investigacions Biomèdiques August Pi i Sunyer (IDIBAPS), Barcelona, Spain; ^2^Institució Catalana de Recerca i Estudis Avançats (ICREA), Barcelona, Spain; ^3^Institute of Psychology, Polish Academy of Sciences, Warsaw, Poland

**Keywords:** bodily perception, embodiment, virtual reality, chronic pain, cortex

## Abstract

Chronic neuropathic pain is highly disabling and difficult to treat and manage. Patients with such conditions often report altered bodily perceptions that are thought to be associated with maladaptive structural and functional alterations in the somatosensory cortex. Manipulating these altered perceptions using body illusions in virtual reality is being investigated and may have positive clinical implications for the treatment of these conditions. Here, we have conducted a narrative review of the evidence for the types of bodily distortions associated with a variety of peripheral and central neuropathic pain conditions. In addition, we summarize the experimental and clinical studies that have explored embodiment and body transformation illusions in immersive virtual reality for neuropathic pain relief, which are thought to target these maladaptive changes, as well as suggesting directions for future research.

## Introduction

Chronic pain is one of the leading health issues worldwide in terms of disability (Vos et al., 2012) and economic cost (Gaskin and Richard, 2012), with an estimated 20% of people affected. Up to about 25% of cases are thought to be primarily neuropathic pain (Bouhassira, 2019), defined as a “pain caused by a lesion or disease of the somatosensory system” (International Statistical Classification of Diseases and Related Health Problems, 2011). Characterized by shooting, burning, or gnawing type pain, and often accompanied by sensory and motor loss, it is highly difficult to manage and treat.

The neurophysiology of chronic pain is extraordinarily complex, highly variable, and very different to that of acute pain. It is associated with changes in the peripheral and central nervous system, including the primary motor and sensory cortices (Apkarian et al., 2009), anterior cingulate and insular cortices, subcortical structures including the amygdala and thalamus (Yang and Chang, 2019), and the spinal cord (Facchin et al., 2021). Cortical changes occur in response to peripheral injury and can be of a neurochemical, structural, or functional nature (Wand et al., 2011). The changes are thought to be largely adaptive and favor functional recovery (Cramer et al., 2005); however, it is thought that they can also be maladaptive and associated with pain and distorted bodily perceptions (Flor et al., 1997; Gracely et al., 2002). The degree of functional cortical reorganization seen in neuropathic pain conditions is highly variable and is thought to depend on the species, age when the injury occurs, the time after the injury has occurred, and the behavioral activity and possible therapeutic regimes after the injury (Moxon et al., 2014). Maladaptive cortical reorganization was long thought to be the primary driver of deafferentation-related pain. More specifically, the theory posits that cortical areas representing the deafferented (either through amputation or nerve transection) body part are freed-up due to a lack of input, and get “invaded” by the neighboring body part, a phenomenon first observed in monkeys (Merzenich et al., 1984). This is thought to generate an input/output error signal that gets interpreted as pain (Harris, 1999). By restoring or normalizing the input, as the theory goes, one can reverse this maladaptive plasticity and thereby reduce pain, and this is the purported mechanism for treatments such as mirror therapy, motor imagery, and phantom motor execution (Moseley and Flor, 2012).

Whilst a significant body of data shows that the degrees of cortical reorganization and pain are correlated (see Vittersø et al., 2022, for a review), this does not necessarily imply causation. Indeed, many subsequent studies have instead demonstrated preservation of the limb area in the cortex (see Makin and Bensmaia, 2017, for a discussion), while more advanced neuroimaging studies have not been able to repeat the purported invasion of the surrounding areas (see Muret and Makin, 2021). Speculation as to the exact neurophysiological mechanisms behind distorted body perceptions and neuropathic pain is largely beyond the scope of this review (for a recent review summarizing the evidence for the maladaptive cortical model, see Vittersø et al., 2022). In any case, the potential perceived bodily distortions associated with these changes include alterations in size or shape, in physical position (altered proprioception), false movements, in levels of ownership or misattributed ownership, in perceived presence or absence of body parts (phantoms and reverse phantoms), distorted interoception (i.e., internal bodily sensations, such as heartbeat), and even feelings of hostility towards body parts, and these distortions seem to be correlated with pain levels.

The association between neuropathic pain, perceptual alterations, deafferentation, and cortical changes is therefore highly complex, with each component being neither sufficient nor necessary for the others to occur. Deafferentation does not always cause pain or perceptual disturbances (e.g., not every amputee reports a phantom limb, and not every phantom limb is painful). Perceptual disturbances can occur in the absence of pain, and in the absence of deafferentation (e.g., with bodily illusions such as the phantom-nose illusion; Ramachandran and Hirstein, 1997). Changes to cortical body representations occur in the absence of deafferentation, pain, and perceptual alterations (e.g., with intense skills-based practice; Elbert et al., 1995). And pain and distorted body image can occur in the absence of deafferentation (e.g., in type 1 complex regional pain syndrome CRPS). Conversely, there are associations between these factors. There are positive correlations between measures of perceptual distortion (perceived limb size, level of tactile acuity) and pain levels in both CRPS and phantom limb pain, for example; and training to improve tactile acuity improves pain levels in these patients (Flor et al., 2001; Moseley et al., 2008). Several studies of amputee patients show that phantom pain severity and the degree of cortical reorganization in the somatosensory and motor cortices are positively correlated (e.g., Flor et al., 1995; Karl et al., 2001; Lotze et al., 2001; Raffin et al., 2016). All of this is suggestive of multiple potential mechanisms that may or may not interact and influence each other.

Although the exact neurophysiological mechanisms remain unclear, treatments that aim to “correct” altered bodily perceptions *via* bodily illusions are thought to be a viable therapeutic avenue and have had some preliminary, if mixed, success (see review by Boesch et al., 2016). However, the relationship between the perceived distortion and the “analgesic” illusion is not always straightforward, and the complex neurophysiology of chronic pain means that even for the same condition these distortions are not always consistently present, and can vary considerably in presentation, which makes their accurate identification an important precursor to effective treatment.

We can get some idea of the general perceptual effects of deafferentation from exploring anesthesia studies, which induce a temporary artificial deafferentation effect. Most of us have experienced dental anesthesia, and the peculiar sensation of facial and lip swelling that comes with it, and many anesthesia studies have shown similar effects of a perceived increase in the size of the affected area (e.g., Türker et al., 2005; Skyt et al., 2015). Healthy subjects report a perceived increase in thumb size following local anesthesia (Gandevia and Phegan, 1999), for example. Further, an illusory elongation of the limb induced by asynchronous stimulation of the virtual body—or disembodiment—has been reported (Perez-Marcos et al., 2018). And in a study of 36 patients undergoing orthopedic surgery with upper limb, lower limb, or spinal anesthetic blocks, Paqueron et al. (2003) found that perceived changes in limb shape and size occurred in more than 30 (83%) of the subjects. Specifically, all 30 perceived their limb to be swollen (an increase in limb width). Eleven patients perceived length changes in the limb (six perceived their limb to be longer, five perceived it to be shorter; by a factor of 2–3 in some cases). Swelling was not perceived as uniform across the limb, however. Most experienced the distal limb to be more swollen, but one patient perceived a normal-sized hand with a swollen forearm, and another reported that he felt his arm and forearm to be enlarged, but not his hand. The timing of these perceptual distortions coincided with the timing of impairment of sensation. Other reported perceptual distortions included a loss of ownership as well as proprioceptive (positional) alterations (i.e., the limb was perceived to be in a different position from where it was in reality). Of note, these distorted perceptions could be corrected when patients visually focused on their limbs (which of course were normally sized). This visually induced correction highlights the high precision weighting given to vision when producing predictions about the state of the body in its environment, and this highlights the therapeutic potential of convincing visual illusions that can be achieved with immersive virtual reality.

We can deduce from anesthesia studies that deafferentation tends to produce a general perception of limb swelling, but with a wide range in other distortions (e.g., in terms of length, ownership, etc.) and no obvious pattern. Acute differences between our top-down self-perceptions and our bottom-up bodily sensations, in particular touch, tend to result in a large prediction error that rises to higher-level brain centers, producing a distorted perception of enlarged size—think of the gap of a recently missing tooth felt with the tongue, or a skin growth or blister felt with the fingers. We speculate that this perceived exaggeration in size may be a protective mechanism that motivates help-seeking behavior when our own body image is acutely altered. Clearly, peripheral neural lesions can cause dramatically altered bottom-up input, and so the resulting potential for prediction error is enormous.

Immersive virtual reality is one such way that bodily illusions, which aim to “correct” these perceptual distortions, can be introduced. Using a combination of a first-person perspective, and visuotactile and/or visuomotor congruent stimulation, the subject can be made to feel as if a limb (Slater et al., 2008; Sanchez-Vives et al., 2010) is their own, in a VR equivalent of the well-known rubber hand illusion (Botvinick and Cohen, 1998). Further, this embodiment illusion can be extended to the whole body (Slater et al., 2009), such that the body of a virtual avatar is felt as one’s own, hereafter termed virtual embodiment.

**Figure 1:**
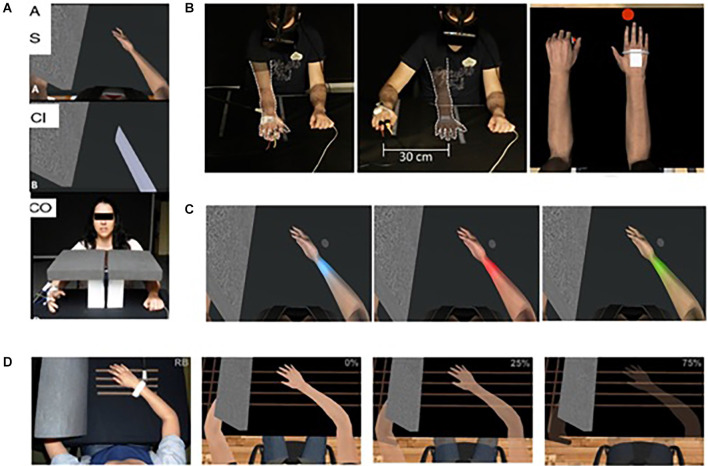
Virtual embodiment experimental setups. **(A)** Martini et al. (2014) showed that the embodiment of a co-located virtual arm is analgesic compared to a non-VR control (in which the arm is hidden; bottom) and a within-VR condition (where a non-corporeal object replaced the virtual arm; middle). Taken from Martini, M., Perez-Marcos, D., and Sanchez-Vives, M. V. (2014). Modulation of pain threshold by virtual body ownership. *Eur. J. Pain* 18, 1040–1048. doi: 10.1002/j.1532-2149.2014.00451.x, © Wiley, used with permission. **(B)** Nierula et al. (2017) showed that the analgesic effect of virtual embodiment relies on co-location of the real and virtual limbs of no more than 30 cm difference. Reprinted from Nierula, B. et al. (2017). Seeing an embodied virtual hand is analgesic contingent on colocation. *The Journal of Pain* 18, 645–655. doi: 10.1016/j.jpain.2017.01.003, © 2017 with permission from Elsevier. **(C,D)** Martini et al. (2013); Martini et al. (2015) showed that manipulation of the appearance of the embodied virtual body can cause an alteration of pain threshold in healthy populations. Used with permission.

Illusions of virtual embodiment have been found to successfully modulate pain threshold in healthy subjects. For example, looking at a co-located and embodied virtual arm has been shown to be analgesic compared to a non-VR control (in which the arm is hidden) and a within-VR condition (where a non-corporeal object replaced the virtual arm; Martini et al., 2014; Figure 1A). This is consistent with experiments showing that looking at one’s own body is analgesic, increasing pain threshold and decreasing cortical responses to pain (Longo et al., 2009). This analgesic effect, or increase in the pain threshold, has been shown to rely on the co-location of the real and virtual limbs, which is feasible in virtual reality but not when using physical fake limbs (Nierula et al., 2017; Figure 1B). The pain threshold in the healthy can be further modulated by transforming the embodied virtual body, for example using color (Martini et al., 2013; Figure 1C) or its consistency/transparency (Martini et al., 2015; Figure 1D), or shape (Matamala-Gomez et al., 2020), opening the door for the use of these strategies in the treatment of chronic pain (for a review, see Matamala-Gomez et al., 2019a).

The purpose of this narrative review is to discuss the type of perceptual bodily distortions associated with various types of chronic neuropathic pain, how they are identified, and whether treatment aimed at reversing these distortions has been successful. By doing so, we hope to provide direction for virtual reality experimentalists and researchers working with people with chronic neuropathic pain; in particular: (1) how to identify those patients that may benefit from immersive virtual reality (VR) treatment focused on addressing/correcting these bodily distortions; and (2) the type of distortion or illusion that could potentially be effective. Other approaches in VR such as mindfulness are also being explored (O’Connor et al., 2022); however, here we focus specifically on bodily illusions.

### Search criteria

The PubMed, EBSCO and Google Scholar, and ResearchGate databases were searched for original research studies published between January 2000 and May 2022 related to two topics: (1) reports of bodily perceptual alterations or distortions in neuropathic pain conditions; and (2) their treatment using immersive virtual reality. The search string “neuropathic pain” AND (“body image” AND “perception”) resulted in 3,300 entries in Google Scholar, 125 in ResearchGate, 10 in EBSCOhost, and 13 in PubMed. The search string “neuropathic pain” AND “virtual reality” resulted in 2,810 entries in Google Scholar, 30 in ResearchGate, 90 in EBSCOhost, and 34 in PubMed. Bibliographies of articles were also hand-searched for other relevant studies. A selective narrative review is provided that does not incorporate a systematic quality assessment of the literature. Studies presented below are those that the authors judged to be most relevant to the topics discussed. After screening the titles of studies selected from the search strategy, only those clearly meeting the inclusion and exclusion criteria were kept. Key inclusion criteria were: (1) quantitative or mixed-methods studies (including case studies); (2) the relevant pain condition; (3) and use of immersive or mixed reality technology; and (4) the intervention/manipulation related to body representations and their distortions. Studies were excluded if the intervention did not include immersive virtual reality (e.g., flat screen), or did not include bodily representation in VR. Three authors split the pre-selected studies and each of them evaluated the eligibility of the abstracts and full texts of the studies assigned to them. The review discusses 19 publications in total, published between 2007 and 2022, including 15 studies on peripheral distortions [phantom limb pain (PLP): 6; CRPS: 8; radiculopathies: 1], and 4 publications on central neuropathic pain (spinal cord injury).

## Neuropathic Pain Conditions and Their Associated Perceptual Distortions

### Peripheral neuropathic pain

#### Phantom limb pain

Phantom limbs have received considerable attention since they were described by the neurologist Silas Weir Mitchell in the 19th century, in soldiers who were injured on the battlefield (Ramachandran and Blakeslee, 1999). They have been intriguing researchers and physicians for decades and a significant amount of literature related to the neural basis of this syndrome has been written. Even so, for people who are not in the field of medicine, it may be difficult to imagine how it is possible to feel a limb that is no longer there, and for that limb to be painful.

The epidemiology of phantom limb sensations (PLS) and phantom limb pain (PLP) varies considerably in the literature. A recent systematic review of 15 studies gave a lifetime prevalence for PLS of 80%, and for PLP of between 76% and 87%, and these sensations occur reasonably quickly after amputation (incidence at 8 days post-amputation 84% and 72% for PLS and PLP, respectively; Stankevicius et al., 2021). Phantom limbs are experienced also by at least 20% of congenital amputees and by 50% of subjects who have amputations before the age of 6 years (Melzack et al., 1997), which is perhaps suggestive of a genetically determined neural hardwiring of our body representation. Phantom limbs can also be experienced in other types of deafferentation injuries such as spinal cord and brachial plexus injuries.

##### Perceptual distortions associated with phantom limbs

PLS usually include specific positions, shapes, or movements of the phantom, feelings of warmth or cold, itching, tingling, or electric sensations. The phantom may be in an awkward position or posture, and the amputee may or may not have control over its movement. In about 25–30% of cases (Flor, 2002; Stankevicius et al., 2021), the sufferer perceives the phantom as telescopically shortened with the distal hand or foot approaching the residual stump, and sometimes even residing within it. PLP is often described as a stabbing, squeezing, cramping, or burning sensation—typical of neuropathic pain—and it is usually intermittent and located in the distal part of the limb (Flor, 2002). A surprising aspect of both PLS and PLP is their precision—pain can often be felt in a specific part of a specific phantom finger, for example; and it is not typically (as is commonly assumed) a vague sensation.

The brain basis for these altered perceptions and pain is, as outlined previously, controversial, with the maladaptive plasticity (Flor et al., 1995), including white matter plasticity (Jiang et al., 2015), retained cortical representation (Makin et al., 2013), and peripherally driven (Birbaumer et al., 1997; Ilfeld et al., 2021) theories all having supporting and refuting evidence. It may be the case that these mechanisms are not mutually conclusive. Altered cortical representations can co-exist with preserved reservations (Muret and Makin, 2021), and some phantom pains may be centrally driven while some may be more peripheral, depending on the individual. The fact that specific phantom limb treatments work very well for some patients and not at all for others, lends credence to this theory. Birbaumer et al. (1997), for example, performed a small study of six upper-limb amputees with PLP. They provided subjects with a brachial plexus block to remove sensory input from the stump, the missing limb and the shoulder, measured their pain levels, and used neuroelectric source imaging to explore cortical organization. Subsequent to the anesthetic block, they found three of the subjects had near-instantaneous pain relief, and their sensory cortical map organization normalized. The other three patients had no pain relief and no corresponding change in their sensory cortical map; and Foell et al. (2014) found that traditional mirror therapy reduced PLP in participants without a telescoped phantom limb by about half, but those with a telescoped limb reported no analgesic effect. The authors posit that the lack of analgesic effect in the subjects with telescoping can be explained by a difference in proprioceptive positioning of the phantom and its visual position in the mirror, such that the mirror hand is not “embodied” (i.e., perceived as the subject’s own).

##### Virtual reality interventions for phantom limb pain

In recent years several studies have investigated the use of immersive VR in the treatment and management of PLP with varying results, although the methodological quality is low and mainly features case series with no controls; indeed, no immersive VR studies met the inclusion criteria in a recent systematic review (Herrador Colmenero et al., 2018), for example. Most of these studies have attempted to reproduce the effects of mirror therapy virtually by transposing movements of the healthy limb to the opposite side. Mirror therapy has been shown to be effective for PLP (Wang et al., 2021), but the type and range of movements that can be performed are restricted since they have to be observable in a mirror. VR removes these restrictions since limb movements can be observed in an entire embodied virtual body seen from a first-person perspective.

Kulkarni et al. (2020) conducted a pilot study of upper limb amputees in which movements of the intact limb were transposed to the position of the amputated limb, seen from a first-person perspective in immersive VR. Nine patients participated in a ball-tracking task in VR, and received three VR sessions over 3 months. A small but non-significant reduction in pain levels was noted in the majority of participants; neither were there any changes in the number of pain episodes nor episode duration. Tong et al. (2020) used a similar mirroring technique in five patients with PLP who had the inability to move their phantoms. Whilst only two patients completed the planned 10 sessions over 6 weeks, all five patients reported a reduction in pain and phantom limb sensations, both post individual session and across all sessions, and that their ability to move their phantom was improved. These results are in line with those of Osumi et al. (2019) who again used a mirror-reverse imaging technique in virtual reality to create the feeling of intentional movement of both limbs (phantom and intact) in a study of 19 amputees. They found the treatment only alleviated certain specific characteristics of the PLP: patients with movement- or positional-related pain and distortions improved more than those with “somatosensory-related pain,” who described typical neuropathic pain sensations such as shooting and burning.

Ichinose et al. (2017) conducted a study with nine participants with PLP, who performed immersive VR neurorehabilitation exercises by touching a target with a virtual representation of their affected limb, the movements of which were again mapped from their intact limb. When they used referred sensation to provide additional illusory touch to the phantom limb, through the application of vibration to the cheek, the analgesic effects were significantly improved compared to a non-tactile condition. This highlights the importance of using congruent multisensory stimulation to improve embodiment over the virtual limb.

Rutledge et al. (2019) conducted a feasibility study in both lower and upper limb amputees. The patients used a pedal machine to drive the movements of a corresponding bi-limbed virtual body seen from a first-person perspective in immersive VR. This visuomotor congruence facilitated the embodiment of the intact virtual limb. They found small reductions in pain and moderate reductions in PLS in all participants.

**Figure 2:**
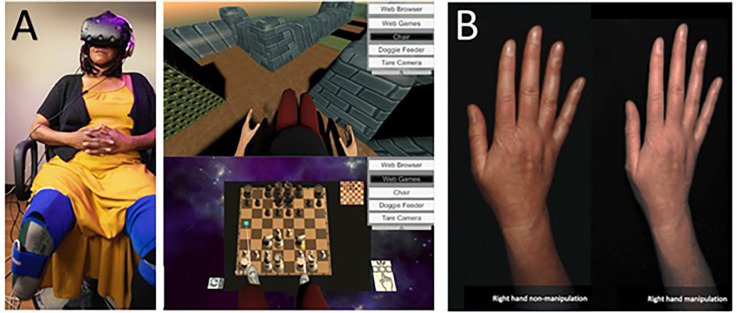
**(A)** Lower limb amputee patient using an immersive system and their view of VR games used in the treatments. Note the visual illusion of having two intact lower limbs seen from a first-person perspective. Taken from Ambron, E. et al. Virtual reality treatment displaying the missing leg improves phantom limb pain: a small clinical trial. (2021) Neurorehabilitation and Neural Repair, 35(12), 1100-1111. doi: 10.1177/15459683211054164. Published October 27, 2021, by SAGE. © The Authors. **(B)** Example of illusory hand manipulation using immersive virtual reality in a patient with CRPS. Taken from Lewis, J. S. et al. (2021). Visual illusions modulate body perception disturbance and pain in Complex Regional Pain Syndrome: A randomized trial. *European Journal of Pain*, 25(7), 1551-1563. doi: 10.1002/ejp.1766. Published March 23, 2021, by Wiley. © The Authors.

Ambron et al. (2021) conducted a small clinical trial where seven transtibial amputees were exposed to repeated immersive VR gaming activities with a non-embodied control condition and an embodied condition that involved playing games by controlling two intact legs (see Figure 2A). They found that six of the seven experienced a pain reduction immediately post-session, with an overall reduction over the course of repeated sessions. PLP reduced by 28% after the control treatment and 40% after the embodied treatment on average post-treatment.

Given the multiple mechanisms that seemingly contribute to PLP, it is unlikely that any single treatment will be effective in the majority of patients, and we can see this clearly in the studies by Foell et al. (2014) and Birbaumer et al. (1997), where there are very clear demarcations between responders and non-responders.

Whilst it is not possible in most cases to disentangle movement and visual effects, in general, the treatments that seem to be of greater efficacy are those orientated at generating a sensation of agency or control over the movements of the phantom—simple visualization of an intact limb appears to be insufficient (Lenggenhager et al., 2014). Whether with mirror therapy, mirror-reverse computer images, or virtual limbs in VR, those therapies that generate the illusion of control and agency over the phantom limb are shown to be partially effective in the reduction of PLP. Those patients with less voluntary control over their phantom, or where it is located in awkward or uncomfortable positions, are more likely to benefit than patients who report primarily neuropathic pain symptoms such as shooting, tingling, or prickling. Thus, determining the characteristics of the patient’s symptoms is important from the outset.

Although no trials yet offer a direct comparison, treatments that include immersive VR in the context of neurorehabilitation (i.e., those that require more attentional and imaginary resources from the patient) may have more potential than the simple visual feedback that takes place in traditional mirror therapy, since the range of possible movements/activities, and environments is limitless in VR, allowing for a more motivational and interesting experience that can be carefully controlled by the experimenter. The rehabilitation can be highly individualized to the patient with appropriate graded progression for example. All of this additionally may improve patient motivation and adherence. It is also important to note that the participants of the studies cited here are adults or senior adults and these conclusions may not apply to children who may need particular consideration. Finally, *a priori* power calculations were not performed for most of these studies (most being small case series or pilots), and larger, better-powered controlled studies are required before therapeutic efficacy can be confirmed. The studies are summarized in Table 1.

**Table 1:** Summary table of virtual reality studies for the treatment of peripheral neuropathic pain.

Authors/year	Study design	Participants/condition	Type of VR intervention	Type of illusion/intervention, plus dosage	Control condition	Time-points	Outcome/results
Ambron et al. (2021)	Clinical trial	Adult lower limb amputees with chronic PLP (*n* = 7)	Immersive VR using HMD, animated limb	Intact virtual limb seen from 1PP, with transposed movements from contralateral intact limb; 5–7 sessions control, 10–12 sessions treatment (done sequentially by all patients)	Non-embodied immersive VR	Post-session and post-treatment) weeks), 1, 4, and 8 week follow up	Mean post-treatment pain of 28% after control treatment, and 40% post-virtual embodiment intervention
Ichinose et al. (2017)	Experimental within-group	Adult upper limb amputees with chronic PLP (*n* = 9)	Immersive VR using HMD, animated limb	Intact virtual limb seen from 1PP, with transposed movements from contralateral intact limb, plus cheek tactile fb, 2–4 days/2–3 sessions per day	As intervention without tactile fb or, fb to intact limb	Pre-post	Mean 16% pain reduction (treatment group) vs. baseline
Kulkarni et al. (2020)	Prospective pilot study, non-controlled	Adult upper limb amputees with chronic PLP (*n* = 9)	Immersive VR using HMD, animated limb	Intact virtual limb seen from 1PP, with transposed movements from contralateral intact limb; 3 × 10 min sessions	None	Pre-post	Mean 23% pain reduction vs. baseline
Osumi et al. (2019)	Experimental within-group	Adult PLP patients: 13 amputees and six brachial plexus avulsion injury (*n* = 19)	Immersive VR using HMD, infrared sensor forhand tracking, agency over the virtual limb	Single session with three tasks (total 20 min): (1) putting the ball into the hall; (2) tracing a figure-eight; (3) loading small blocks. Control the virtual phantom limb with the intact limb movements	None	Pre-post	Mean 59% pain reduction vs. baseline
Rutledge et al. (2019)	Experimental within-group	Males adult veterans with PLP: upper (1) and lower (13) amputees (*n* = 14)	Immersive VR, HMD, motion sensor, and pedaler	Mirror therapy paradigm. Intact virtual limb seen from 1PP using a basic bicycle pedaler on the floor (lower limb amputees) or on a table (upper limb amputee). Duration and frequency of the VR intervention was variable. 10 participants completed 1 baseline visit and a single treatment session. four participants completed multiple sessions (5/ 12/ 14/ 28) either in the laboratory or at home.	None	Pre-post	Near complete elimination of phantom sensations Small reduction in pain (individual values NR)
Tong et al. (2020)	Experimental within-group	Adult upper limb patients with chronic PLP (*n* = 5)	Immersive VR using HMD, animated limb	Intact virtual limb seen from 1PP, with transposed movements from contralateral intact limb, 10× sessions over 6 weeks	None	Pre-post	Mean 57% pain reduction vs. baseline
Chau et al. (2020)	Pilot longitudinal study with one group	Adult patients with CRPS (*n* = 8, 6 completed)	Immersive VR with HMD	Ten sessions of exercises in a virtual kitchen with hand movements tracked; mirrored hand movements options if holding the controller was too painful	none	Pre-post each session	Four of six participants reported subjective pain reduction and daily functioning improvement, no change in objective pain scales, low correlation between subjective and objective pain scales
Hwang et al. (2014)	RCT	Adults with CRPS (*n* = 39)3 conditions, each *n* = 13	Recorded video of movements seen from 1PP displayed in HMD	Virtual body swapping with mental rehearsal. Single session	Watching movement only, mental rehearsal only	Pre-post	No pain decrease in any of the groups, BPD improved only in virtual body swapping with mental rehearsal group
Jeon et al. (2014)	RCT	Male adults with CRPS I (*n* = 10)	Immersive VR using HMD, recorded video	Body swapping illusion, with explicit motor imagery; 3 m 20 s long video clip played 2×, training session max. 10 min long	Body swapping illusion, passive observation	Pre-post	No difference in pain between groups (absolute values NR). BPD improved in the treatment group, not controls
Lewis et al. (2021)	RCT	Forty-five patients with CRPS and BPD, single exposure: experimental group *n* = 23, control group *n* = 22; repeated exposure subgroup: *n* = 21 and *n* = 18 respectively	Mediated virtual reality system MIRAGE	All participants: 1 session × 1 min, repeated exposure subgroup: five sessions (2 weeks); altered/Unaltered image of the affected limb, Altered image according to the patient’s description of the desired hand’s look	Unaltered image of the limb	Pre-post intervention in each session, pre-post study in the repeated exposure group	Pain and BPD reduction post VR exposure, maintained at f/u; mean 11% reduction in pain intensity
Matamala-Gomez et al. (2019b)	Experimental within-group study	Adults with CRPS (*n* = 10) or with PNI (*n* = 9)	Immersive VR with HMD	Virtual embodiment and manipulation of avatar to change limb size (three levels) and transparency (four levels)	Non- manipulated avatar condition for size and transparency	Pre-post	Mean overall post-exposure pain reduction of 50%. Increasing transparency reduced pain in CRPS and increased pain in PNI. Increased size increased pain in CRPS only.
Sato et al. (2010)	Open-label case study	CRPS adults (*n* = 5)	Desktop virtual reality mirror visual feedback	Visual motor therapy with a Cyberglove for finger motion simulation and FASTRAK for arm movement simulation, 1 session/week, 5–8 sessions	None	Each session Pre-post	4/5 patients had >50% reduction in pain intensity
Solcà et al. (2018)	Crossover double-blind study	Adult patients with CRPS (*n* = 24)/Healthy controls (*n* = 24)	Immersive VR powered by biofeedback	Visual heart rate feedback (flashing in synchrony/asynchrony) presented on a virtual depiction of the affected limb, within-subject design, two conditions, Each repeated three times consecutively, single stimulation lasted 90 s	Hand flashing asynchronous to heart beat	Pre-post block, Pre-post stimulation	Sig. pain reduction (−1 point), increased strength (0.75kg), modulation of heart rate variability only in experimental conditions (−3 ms) and healthy controls (individual values NR)
Won et al. (2015)	Two descriptive between-subjects pilot studies	Pediatric patients (between 13 and 17 years old) with CRPS (*n* = 4)3 conditions: (1) normal condition (legs in a one-to-one relationship); (2) extended condition)leg movements were increased by afactor of 1.5); (3) switched condition (legs controlled the avatars’ arms)	Immersive VR using HMD, spatialized audio feedback, haptic feedback	Interaction in 1PP with a sequence of balloons at different distances. When the balloon was hit, audio feedback was provided and the floor vibrated slightly. 6 × 5 min sessions.	None	Pre-post	Subjectively, better treatment adherence and motivation, less complaints about pain during treatment
Solcà et al. (2021)	Randomized, within group study	Patients with chronic radicular pain (*n* = 15)	Immersive VR with HMD	SCS with virtual embodiment; area of altered sensation from SCS highlighted on the virtual body, single session 7.5 min	Incongruent SCS-VR and VR alone	Pre-post	Mean pain reduction of 44% in the treatment group (23% in incongruent SCS-VR, 3% in VR only)

NR, not reported; BPD, body perception disturbance; CRPS, complex regional pain syndrome; SCI, spinal cord injury; SCS, spinal cord stimulation; PLP, phantom limb pain; 1PP, first-person perspective; TDCS, transcranial direct current stimulation; fb, feedback; sig, significant; Nsig, not significant.

#### Complex regional pain syndrome

Complex regional pain syndrome is a multifactorial disorder of the upper or lower limb characterized by allodynic pain, swelling, focal dysautonomia including vasomotor instability, skin color/temperature abnormalities, and hyperhidrosis. It is usually preceded by an injury that necessitates a period of immobilization, but symptoms persist even after healing appears complete. In the acute phase the limb becomes sensitive and swollen with an elevated temperature, before progressing to a chronic phase in which the inflamed appearance has resolved, limb temperature decreases, but pain and disability remain (Maihöfner, 2014). Along with temperature changes, patients often report neglect-like symptoms, both cognitive (the limb feeling foreign) and motor (directed mental and visual attention needed to move the limb; Galer and Jensen, 1999), as the impaired spatial perception results in a spatial-dependent modulation of thermoregulation and bodily ownership over the affected limb (Moseley et al., 2012). The pain is often described as strong and burning, and does not follow a dermatomal or peripheral nerve distribution. In addition, patients may report a desire to amputate the limb. These “neglect-like” symptoms indicate an important role of attention and changes in the motor and parietal cortex (McCabe et al., 2009). Patients commonly mention a large discrepancy between how the affected limb looks and how it feels, including a sensation that the limb is bigger, hotter, or heavier than it is in reality (Lewis and McCabe, 2010). The literature distinguishes two types of CRPS, with no obvious neural damage (type 1) and when nerve damage can be identified (type 2), although this classification may be only apparent (Oaklander et al., 2006), and here we will consider both types together.

CRPS is thought to be relatively common, but the reported prevalence in the literature varies from 1% to 37% in distal radius fracture for example (Ortiz-Romero et al., 2017). It is more common with upper limb injuries and in females. Immobilization is a significant risk factor with 47% of 134 CRPS sufferers in one retrospective case study having a history of medically imposed limb immobilization (Allen et al., 1999).

The pathophysiology of CRPS is a highly complex interaction between multiple systems, including peripheral and central sensitization, autonomic and immunologic dysregulation, genetic influences, and psychological stressors. There is also a significant body of literature demonstrating the existence of cortical reorganization in CRPS, including referred sensations between stimulated body parts (Maihöfner et al., 2006), and disturbances of the somatosensory and motor systems.

Current treatments include bisphosphonates, neuropathic and anti-inflammatory medications, spinal cord or dorsal root ganglion stimulation, and physical therapy modalities including mirror therapy or graded motor imagery (Shim et al., 2019). The efficacy of these treatments is variable, and a recent systematic review concluded that although there appears to be a therapeutic role for these interventions, confirmatory RCTs are warranted (Duong et al., 2018). For a review of literature published before 2010, see Swart et al. (2009) and McCabe et al. (2009). Here, we will focus on work on body distortions in CRPS from last decade.

##### Perceptual distortions in CRPS

###### Limb perception and representation

One of the most common symptoms in CRPS is the distorted perception of the affected limb’s size. In a task where schematic drawings were compressed or expanded from 85% to 120% of the original size, patients with CRPS claimed their affected limb was bigger than it really was, compared with healthy controls (Peltz et al., 2011). Moreover, there was a positive relationship between the overestimation and disorder duration, neglect-score, and decreased tactile thresholds, which suggests a disruption in the integration between motor processes and the body schema (a dynamic representation of the human body based on its localization in space, Biran et al., 2022) in the central nervous system (Peltz et al., 2011). Lewis and Schweinhardt (2012) studied the relationships between pain levels, body perception disturbance, and tactile acuity. In a controlled observational study, patients who reported greater pain had more extensive body perception disturbances, measured with the Bath CRPS body perception disturbance scale (Lewis and McCabe, 2010). Moreover, patients with stronger body perception disturbance were characterized by worse tactile acuity in a two-point discrimination task, and a longer duration of symptoms was positively related to greater body perception disturbances, confirming the results obtained by Peltz et al. (2011). The authors indicated the involvement of the central nervous system, where the primary somatosensory (S1) and posterior parietal cortices play a role in body image disruption in CRPS patients. They suggested that the affected limb’s cortical representation shrinks, while the representation of the healthy limb remains unaltered. This finding is not without controversy, however. Di Pietro et al. (2015) used functional magnetic resonance imaging (fMRI) to measure the S1 representation in patients with CRPS and healthy controls. The measure of S1 representation was based on the distance between digits one and five activation maxima for both hands. The results suggest that it is actually the healthy limb’s representation in S1 that becomes enlarged and not the representation of the affected limb that shrinks. This may be an adaptive change related to the compensatory increased use of the unaffected limb. Mancini et al. (2019) question previous work on S1 changes as being related to pain, arguing that the S1 size estimation by measuring the Euclidean distance between activation loci of the thumb or index finger and of the little finger is indirect and imprecise. Instead, they used fMRI to measure the somatotopy of the full hand and found that the size of the cortical representation of the affected limb was mostly comparable to those of a healthy limb or with healthy controls. Their data corroborate the effect of disease duration, but they found no relationship with pain intensity, pain sensitivity, and severity of the physical disability. They conclude that the rationale for interventions aimed at restoring somatotopic representations to treat pain is questionable.

###### Body and peripersonal space

There is a growing interest in the perception of external space in CRPS, and may offer a better explanation of neglect-like symptoms than purely cortical mapping alterations (Moseley et al., 2013). For example, Reid et al. (2016) demonstrated that the somatosensory perceptual distortions are rooted in cognitive processing, and not in altered peripheral coding or spinal transmission of somatosensory inputs. They conducted three studies. In the first, patients performed left/right judgments of pictured hands or feet and of two-dimensional line-drawn letters. The second consisted of temporal order judgments, while the final study consisted of bisection tasks. Longer reaction times when the hand/foot image was presented on the side of the monitor ipsilateral to the affected limb were noted, and there was also a bias in tactile processing toward the healthy side. Most interesting were the results of the bisection judgments on body parts with judgment 14 ± 2 mm from the true midline toward the healthy side. Since the spatial perception of auditory stimuli (outside the body space) was not impaired, the authors claimed that spatial cognitive distortions in CRPS are related to body-related information, and not to the external space perception. Conversely, Filbrich et al. (2017) suggest that this is not completely true. In the visual temporal order judgment tasks, visual stimuli directly surrounding the affected limb were perceived significantly later than stimuli presented next to the unaffected hand, suggesting possible visual distortions in the perception of external stimuli around the affected limb.

Brun et al. (2019) demonstrated a disturbed sense of limb position and alterations in the sense of active limb movement in CRPS patients (compared with healthy controls), in a study utilizing a robotized exoskeleton and a 2D virtual environment in which a virtual upper limb projection was displayed. Nonetheless, these phenomena are not related to the perception of the affected limb. The authors suggest that the sense of limb position and limb movement depend on online sensorimotor integration (therefore, construction of the body schema), while body image distortions in CRPS patients are directly related to the “neglect-like” symptoms.

In contrast, Halicka et al. (2020) show no evidence of systematic spatial biases in visuospatial attention to, or representation of, the side of the space close to the affected limb. Moreover, data do not corroborate the concept of directional slowing of movements toward the affected limb consistent with motor neglect. Nonetheless, the authors reported that CRPS patients initiated and executed movements, with both healthy and affected limbs, more slowly than healthy controls (Of course, people in pain do tend to move more slowly in general, often for reasons of hypervigilance and kinesiophobia, so this is not necessarily reflective of cortical alterations). Moreover, there was no evidence of changes in spatial cognition, measured by the strength of the relationships between the size of spatial biases and the severity of CRPS symptoms. They confirm that there is a direct link between pain levels and CRPS perceptual symptom severity and that patients present with subjective body perception disturbance alongside motor impairments. They therefore recommend a treatment focused on correcting body representation and motor functioning.

In a recent study, Vittersø et al. (2019) investigated the updating of bodily and spatial representations in patients with CRPS. Compared with healthy controls, patients demonstrated more malleable representations. Authors suggest that in CRPS, body and peripersonal space perception is less stable than in healthy people and indicates alterations in neuropsychological processing of the affected side and not specifically the affected limb.

Notably, recent behavioral and EEG evidence indicates that in CRPS both interoception and exteroception may be disturbed. Solcà et al. (2020) observed that CRPS patients scored lower than age-matched healthy controls in a heartbeat counting task. The behavioral result was replicated in an EEG study, with significant suppression of the heartbeat-evoked potential (HEP) in CRPS patients (compared with healthy controls) observed at the predicted location and in the specific time window. Furthermore, there was a correlation between the amplitude of the HEP and performance in the heartbeat counting task. Together, these results indicate that changes in the neural body representation include both exteroceptive and interoceptive bodily signals.

##### Virtual reality interventions for CRPS

Several studies have investigated the use of VR as a potential therapeutic modality (summarized in Table 1). Sato et al. (2010) used VR mirror visual feedback therapy to reduce pain. The system consisted of a desktop application and a special glove to simulate the finger movements of the unaffected limb on the affected side. The training included sequences of movements while focusing on the motion of the virtual hand on the monitor. Two patients ended the training after five (out of eight) sessions, and overall pain reduction for four out of five patients was over 50%. Although preliminary, the study offered evidence that a VR-based intervention in which “normal” movements of the affected limb were experienced, may have analgesic potential.

As discussed above, immersive VR allows the user to embody a virtual body in place of their own (body swapping). In an early implementation of the full body swapping illusion, Jeon et al. (2014) demonstrated that watching a virtual body swapping training video in a HMD reduced body perception disturbance (BPD) more efficiently than the control condition. BPD is defined as the perceived alteration of the CRPS-affected body part while regarding the rest of the body as normal and includes such symptoms as a sense of disownership, lack of attention to the limb, distorted mental visualization, and different perception of size, shape, weight, pressure, or temperature of the impaired limb (Lewis and McCabe, 2010). Although there was no analgesic effect, the body swapping illusion in virtual reality is a promising direction for CRPS treatment, since generally pain levels and degrees of BPD tend to be correlated (Lewis and McCabe, 2010). Hwang et al. (2014) also explored a body-swapping intervention, in which 39 patients were assigned to one of the three experimental groups: the “virtual body swapping with mental movement rehearsal,” the “watching movement only,” and the “mental rehearsal only.” However, only the body-swap group demonstrated a significant improvement in BPD and there was no significant pain reduction in any of the conditions.

The body swapping illusion in VR was also applied to pediatric patients in two pilot studies (Won et al., 2015). Here, four adolescent participants were tasked with hitting their virtual leg with a balloon. A successful hit was accompanied by audio (a “pop” sound) and tactile feedback (delicate vibration of the floor). Study 1 (two patients) compared two conditions; in the control condition, the virtual leg moved in a one-to-one relationship with the real leg, while in the experimental condition, the virtual leg’s movement was amplified by 50% of the real leg’s movement. In study 2 (two patients), a third condition was added where the patient controlled their arm with their leg. The preliminary results based on six sessions confirm the safety and feasibility of the VR treatment, together with high engagement of patients. The authors did not draw any conclusions on efficacy for reducing the pain, but do suggest the treatment hold promise for increasing mobility.

Solcà et al. (2018) presented a much more technologically advanced and efficient digital therapy based on heartbeat-enhanced virtual reality (HEVR), which combines high control over the procedure in prolonged and repeated doses and an automatized integration with existing treatments, at the same time reducing the burden of repeatable long training sessions for both the patient and the therapist. During HEVR, participants looked at their virtual hand flashing either synchronously or asynchronously with their online-detected heartbeat. The synchronous flashing resulted in a significant pain reduction and strength increase, which was not observed in the control condition, which differed only in the cardiovascular illumination of the virtual hand. The intervention was based on a modulation of the central body representation using multisensory stimulation. In particular, the analgesic effect was a result of the partial overlap and mutual connections between central pain representations and the body matrix, benefiting from the anatomically closely related pathways of pain and cardiac signal processing.

A pilot study by Chau et al. (2020) used a virtual kitchen environment to simulate real-life activities such as washing hands, tossing a article airplane, assembling a sandwich, sorting dishware, and arranging utensils. Six out of eight participants completed the study and four of them reported a subjective pain reduction and improvement in daily functioning. Nonetheless, objective pain scales did not corroborate this effect.

Another application of virtual bodily illusions was recently published by Lewis et al. (2021). In a randomized trial study, CRPS patients embodied a digital image of their affected hand. In the experimental group, the appearance of the hand was altered in real-time according to patients’ suggestions so that it matched the patient’s desired appearance (in terms of shape, size, and/or color), while in the control group, the image was unaltered (see Figure 2B). The intervention brought pain and body perception disturbances reduction after a single exposure (compared to controls) and was sustained after 2 weeks in the follow-up sub-group.

**Figure 3:**
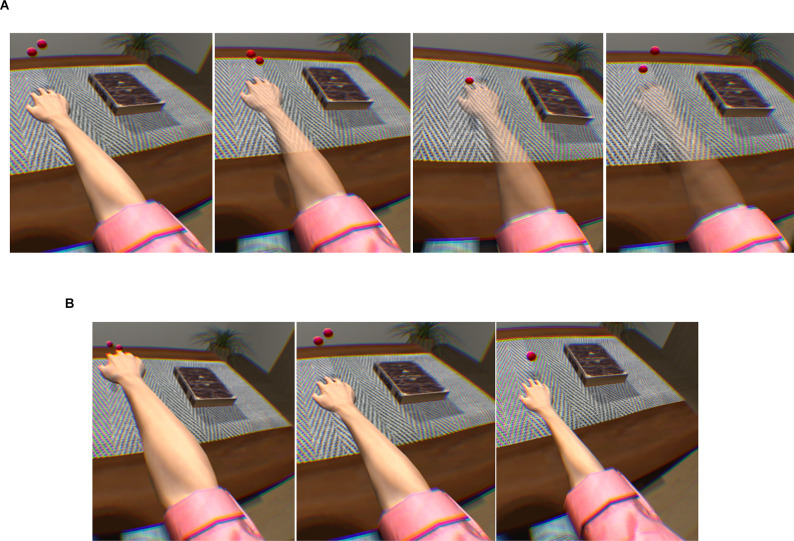
Transparency and size illusions of an embodied virtual arm. **(A)** Virtual arm illusion showing arm at 0%, 25%, 50%, and 75% transparency. **(B)** Virtual arm illusion showing enlarged, normal, and reduced size arm. Note the use of bouncing red balls in which vibration feedback is provided when the balls touch the skin. This visuotactile congruence stimulation helps induce the embodiment illusion. Reprinted from Matamala-Gomez, M. et al. (2019b). Decreasing pain ratings in chronic arm pain through changing a virtual body: different strategies for different pain types. *The Journal of Pain* 20(6), 685–697. © 2019, with permission from Elsevier.

Matamala-Gomez et al. (2019b) compared the impact of varying size and transparency levels of the illusory embodied virtual body in two types of chronic pain patients—those with type I CRPS against those with peripheral nerve injury. They modified the visual aspect of the patient’s embodied virtual arm, comparing three levels of size and four levels of transparency (see Figure 3). All seven conditions taken together decreased post-exposure pain ratings by half, however, not all visual manipulations had the same impact: increasing virtual arm transparency was effective at decreasing pain in CRPS but not in peripheral nerve injury while increasing virtual arm size increased pain ratings in CRPS only. Overall, different etiologies of chronic pain seemed to require different strategies in order to be analgesic.

CRPS is a poorly understood and difficult to treat condition. In the last decade, several studies investigated the usefulness of IVR for CRPS treatment. Most of them were pilot studies or case series, with low numbers of participants and limited controls. Investigated treatments included virtual body aspect alterations and movement observations/performance. There is some evidence of reduction in BPD and pain, and for improvement in function; nonetheless, pain reduction was not universal and was not always linked to BPD. A high selective efficiency in pain reduction and strength increase was shown by a study combining virtual embodiment with real-time heart rate visualization. Overall, IVR-based interventions are feasible, safe, and engaging for patients of all ages and genders, but more studies with bigger sample sizes and improved methodology are required to confirm their usefulness in pain treatment.

### Radiculopathies

Chronic low back pain has been associated with central neuroplastic changes, which may be driven by maladaptive beliefs and behaviors, leading to distorted perceptions such as altered dimensions and postures, and a perceived lack of movement control (Wand et al., 2016). Lumbar radiculopathy, in which the spinal nerve root is compressed (usually by a herniated disc) causing significant low back and leg pain, weakness, and paresthesia, is a less common cause of low back pain (5–10% of cases). The mechanism is thought to be a combination of neuropathic and nociceptive pain, due to the compression and the peripherally sensitizing inflammatory process, respectively. Over time, these mechanisms are thought to interact (e.g., prolonged inflammation causing significant demyelination). There is a paucity of evidence relating radiculopathies* per se* with central changes and therefore altered perceptions. Radiculopathy patients can present with distorted perceptions, but the likely culprit is thought to be maladaptive beliefs and resulting behaviors and not deafferentation itself; for example, belief that pain signifies significant damage and the low back needs protection may induce slow, stiff, guarded movement strategies resulting in altered sensorimotor processing and cortical changes, just as is the case for non-radicular spinal pain (Wand et al., 2012).

Just one immersive VR intervention for radicular pain has been reported in the literature. In a within-subject experimental study of 15 patients with lumbar radiculopathy following failed lumbar surgery, Solcà et al. (2021) used a virtual embodiment illusion to “highlight” the areas of leg sensation felt when patients received therapeutic spinal cord stimulation. They found significant short-term improvements in pain compared to VR-only and incongruent visual highlighting conditions.

Generally speaking, we speculate that VR interventions that improve confidence and help strengthen the belief that the back is strong and stable, and that try to restore normal thoughtless, fearless movement are likely to be of benefit (just as such treatments based in reality do). Preliminary pilot studies that have explored the virtual embodiment of strong, fit backs show promise for patients with non-radicular chronic low back pain (Nishigami et al., 2019; Harvie et al., 2020).

### Traumatic avulsion

Traumatic nerve avulsion injuries, especially of the brachial plexus, are common in road traffic accidents and (less commonly) in contact sports such as rugby or mixed martial arts. Sometimes these patients can develop painful phantom limbs, due to the massive deafferentation associated with the injury. Melzack and Wall (1996) describe one such patient whose left arm was completely paralyzed but intact; however, he described a phantom left arm that was placed across his chest with a tightly clenched fist and the nails digging into his palm. These injuries are associated with cortical changes. In a study by Pourrier et al. (2010), 10 patients with traumatic upper limb nerve injuries (and subsequent repair surgery) underwent sensory testing. Three were found to have referred sensations from the face to the hand (i.e., when their face was touched, they felt a corresponding sensation in their hand), highlighting the somatotopic reorganization of the sensory cortex in response to the injury.

We found only one study that has explored the use of immersive VR for this type of injury. The previously mentioned study by Matamala-Gomez et al. (2019b) showed that, in general, patients with peripheral nerve injury can readily embody a virtual arm in immersive virtual reality, and that this appears to be analgesic in the short term. However, specific manipulations such as size or transparency did not correlate with the induced analgesia—indeed, increasing transparency appeared to worsen patients with peripheral nerve injury.

## Central neuropathic pain

### Spinal cord injury

#### Perceptual distortions in spinal cord injury

More than half of the patients with spinal cord injury (SCI) suffer chronic neuropathic pain syndromes at or below the level of injury (Burke et al., 2017). While pain may result from direct damage to the spinothalamic tract, damage to the tract does not always result in pain. It is thought that ongoing activity in residual spinothalamic pathways plays a key role in the maintenance of central pain in SCI (Wasner et al., 2008). In addition, the reorganization of the somatosensory systems and changes in body representations related to the massive deafferentation associated with SCI (Jutzeler et al., 2015; Osinski et al., 2020; Vastano et al., 2022) are thought to play a role in both the development of pain and perceptual alterations, although the degree to which the cortex reorganizes is highly variable (Moxon et al., 2014). Vastano et al. (2022) provide a comprehensive overview of cortical changes following SCI, their associated perceptual alterations, and the use of multisensory bodily illusions to induce pain relief. Most clinical reports on perceptual alterations come from single case studies and small case series; however, Scandola et al. (2017) published a much larger case series of 49 patients with spinal cord injury. They conducted extensive interviews and collected questionnaires concerning a variety of body related feelings, measured the presence of visceral, musculoskeletal, and neuropathic pain, and personality variables. They then categorized the resulting bodily perceptual distortions into aix categories:

1. Body parts disappearing
2. Involuntary motion, including feelings of muscular fatigue
3. Mislocalization (body parts in the wrong place)
4. Misoplegia: aversive feelings towards a body part
5. Disownership-like feelings
6. Somatoparaphrenia: the sensation that body parts belong to someone else, or are detached from the body.

Of these only disownership and somatoparaphrenia seemed to be correlated. Body part mislocalization was the most common sensation, occurring in all of the tetraplegic patients with complete injury. Feelings of disownership and somatoparaphrenia were more frequent in paraplegic patients (especially those with incomplete lesions) than in tetraplegic patients. They found that the presence of musculoskeletal pain and misoplegia were correlated; and that neuropathic pain was negatively correlated with perceptions of involuntary motion (Scandola et al., 2017). Interestingly, in only four patients were body size alterations noted. In contrast, Fuentes et al. (2013) compared dimensional body image perceptions in 42 tetraplegic and paraplegic patients and 18 healthy controls and found that paraplegic and tetraplegic patients both perceived their torso and limbs as elongated relative to their body width, as compared with healthy controls. Crucially, however, the spinal level of injury made no difference. The authors, therefore, question the link between sensorimotor loss *via* deafferentation and body image, suggesting instead that the body image changes seen could instead be reflective of postural changes and being unable to stand or walk (Fuentes et al., 2013). Such results do seem to contradict the findings from amputation and anesthesia studies, which implicate sensorimotor loss and subsequent cortical alterations as the primary mechanism of body image distortion. However, the subjects had to locate their various body parts with an anchoring stimulus on a screen, a task that requires visuospatial and motor imagery skills, and therefore did not isolate perceived body image.

Other studies have also shown perceptual alterations in SCI. Arnhoff and Mehl (1963) showed that paraplegic patients overestimate their shoulder width. Conomy (1973) showed a perceived increase in foot and leg size in tetraplegic and paraplegic patients. Melzack (1990) describes the case of a quadriplegic patient who experienced bilateral phantom legs that were continuously positioned at 90 degrees—when lying on his back his phantom legs protruded straight up into the air! The VR interventional studies for neuropathic pain in SCI are summarized in Table 2.

**Table 2:** Summary table of virtual reality studies for the treatment of central neuropathic pain.

Authors/year	Study design	Participants/condition	Type of VR intervention	Type of illusion/intervention, plus dosage	Control condition	Time-points	Outcome/results
Moseley (2007)	Experimental within-group	Adult SCI patients with chronic pain (*n* = 4)	Mirror/recorded video split screen	Virtual walking observed in a virtual mirror, daily 3 weeks	Guided imagery, watching a film	Baseline, 3 weeks, 3 months	Mean 53% reduction in pain intensity at 3 weeks, 43% at 3 months from baseline
Pozeg et al. (2017)	Randomized, repeated-measures study	Adult SCI patients with chronic pain (*n* = 20) Healthy controls (*n* = 20)	Immersive VR with HMD, real-time video	Virtual leg illusion, Virtual body illusion Single session	Healthy controls (*n* = 20), asynchronous tactile stimulation	Pre-post	Weaker embodiment in SCI vs. healthy controls Mild analgesia for both conditions post- treatment (individual values NR)
Richardson et al. (2019)	RCT	Adult SCI patients with chronic pain (*n* = 59)	3D video	Virtual walking from a 1PP Single session	Virtual wheelchair mobilization from a 1PP	Pre-post	NSig difference between groups. Post sig. reduction pain intensity (individual values NR)
Soler et al. (2010)	RCT	Adult SCI patients with chronic pain (*n* = 40)	Mirror/recorded video split screen, combined with TDCS	Virtual walking observed in a virtual mirror; 10 sessions/2 weeks	Sham TDCS, video images unrelated to gait	Baseline; 2, 4, 12 weeks	Sig reduction in overall pain intensity post-treatment in DCS+illusion groups, maintained at follow up (individual values NR)

#### Virtual reality interventions for SCI-related pain

Current treatments for SCI-related neuropathic pain include pharmacological, surgical, electrotherapeutic, physiotherapeutic, and psychological treatments, with only marginal overall benefit. There is also some recent limited, but promising evidence for motor imagery interventions (Moro et al., 2021). Austin and Siddall (2019) published a recent narrative review of virtual reality treatments for SCI pain. Only nine small and low-quality studies met the inclusion criteria, and while there are some promising results, they conclude further high-quality studies are required. Since below-level neuropathic pain is thought to be primarily supraspinally driven, it follows that VR interventions that primarily target cortical representations (as opposed to short-term distraction mechanisms) are more likely to be more successful for this type of pain over the long term (Austin and Siddall, 2019).

A number of studies have explored the use of a virtual walking illusion. Moseley (2007) explored the use of a virtual walking illusion in five patients with incomplete paraplegia. The patients visualized themselves walking while observing a film/mirror setup that gave the illusion they were walking. He found that pain was significantly reduced in four of the five patients (all of which had at-level pain); the other patient (with below-level central pain) experienced an increase in pain severity. The author suggests the difference in responses may be related to differences between central and peripheral pain syndromes (the four responders had cauda equina lesions which are technically peripheral lesions and not spinal cord lesions *per se*; the non-responder had a T12-level injury, considered central). Analgesia was maintained at 3 months following a subsequent 3-week daily treatment in the responders. Soler et al. (2010) used a similar setup to Moseley (2007) but combined the VR treatment with transcranial direct current stimulation (TDCS). While greater benefits were achieved with a combination of TDCS and virtual walking, virtual walking alone still produced significant reductions in pain severity; however, this was not maintained at follow-up. Richardson et al. (2019) compared virtual walking to virtual wheelchair pushing and found significantly greater pain reduction for the virtual walking setup, irrespective of at-level or below-level pain. These apparent differences between the results of these virtual walking studies may therefore be explained by differences in set-up, dosage, level of pain, level of injury, and degree of injury.

Pozeg et al. (2017) examined changes in body ownership and neuropathic pain in paraplegic patients in a randomized repeated-measures controlled study. They used visuotactile congruent stimulation to induce ownership over a virtual leg in paraplegic patients and controls. The SCI patients showed a small but significant pain reduction, but less embodiment than: (a) the healthy controls; and (b) when other body parts were embodied. These studies are summarized in Table 1.

Overall then, the huge variability in potential perceptual alterations that may result from SCI, together with a wide variety in individual responses to treatment, suggest that: (1) careful individual screening should be performed to try to determine the presence and type of any perceptual alterations; and (2) that individually tailored illusions, which can be relatively easily implemented in VR, are more likely to be of benefit.

## Other conditions

The following conditions are those in which distorted bodily perceptions have been reported in the literature, but where there have not been specific VR interventions aimed towards addressing these perceptions, or where the perceptual interventions were not VR based.

### Peripheral compression neuropathies: carpal tunnel syndrome

Carpal tunnel syndrome (CTS) is the most common peripheral compression neuropathy. Some studies have found enlarged and/or blurred cortical representations in contralateral S1 for the digits hand in patients with CTS (Druschky et al., 2000; Tecchio et al., 2002; Napadow et al., 2006), and this is thought to manifest clinically as sensory discrimination accuracy compared with healthy controls (Maeda et al., 2014). A study by Schmid and Coppieters (2012), for example, found that patients with unilateral CTS had impaired left/right judgment (i.e., the ability to determine whether a viewed hand is on the left or right side) of the painful arm/hand. The deficit was similar to that reported for more severe conditions such as CRPS and phantom limb pain. Perceived size differences have not been reported. Yoshida et al. (2020) used magnetoencephalography and a static and moving two-point discrimination task to investigate the mechanism underlying the sensation of pain beyond the median nerve territory of the affected hand. The study proved an activation of bilateral sensorimotor areas in sites other than the hand and high activation of the inferior parietal lobule in the left hemisphere. Overall, results suggested that pain in extra-territorial areas may be linked to a dysfunction of spatial cognition in CTS patients. No studies have explored whether addressing these perceptual deficits using immersive VR has any effect on symptoms in CTS; however, a single case report that explored the use of explicit motor imagery had positive results (Anderson and Meyster, 2018), therefore therapeutic movement using virtual embodiment may be of interest to explore.

### Post-stroke pain

Central post-stroke pain is common, affecting between 11% and 55% of patients following a stroke (Leijon et al., 1989; Andersen et al., 1995; Vestergaard et al., 1995). It seems to occur where the infarct affects some of the central nociceptive processing pathways (spino thalamo cortical pathways), without actual stimulation of peripheral nociceptors (Treister et al., 2017). Head and Holmes (1911) put forward a disinhibition theory which suggested that injury to these sensory pathways leads to a compensatory overactivation within the thalamus.

Clinically, central post-stroke pain is often missed, since pain is quite common in stroke patients for a number of other reasons such as the development of CRPS, joint subluxation, and pain associated with spasticity. Central post-stroke pain onset can be variable but most often begins 1–3 months post-stroke with most patients affected before 6 months post-stroke (Kumar and Soni, 2009). It is well documented that stroke is associated with a number of perceptual alterations, both common (e.g., neglect, visuospatial problems) and relatively unusual (phantom or supernumerary limbs), but these are thought to be primarily a result of the infarction itself and not a subsequent result of any associated pain, although the relationships may be complex. In a study of 50 post-stroke patients, Antoniello et al. (2010) noted phantom experiences in just over 50%, with either a positional phantom (*n* = 22), a kinesthetic phantom (*n* = 14), or a mix of the two (*n* = 10). Interestingly, only in one patient was the phantom perceived as painful and the timing of the mixed or kinesthetic phantom (mean 27 months) does not coincide with the typical time to development of post-stroke pain. More common perceptual problems such as neglect tend to occur in the acute phase of stroke, and perceptual distortions are therefore unlikely to be driven by the same mechanisms as central post-stroke pain, which is usually of a much slower onset. The phenomena of asomatognosia and somatoparaphrenia (disownership and misattributed ownership, respectively) in stroke is well documented, and studies that have explored the use of embodiment illusions in patients with these conditions found that they are stronger illusory effect on the affected vs. the unaffected side (van Stralen et al., 2013) and vs. healthy controls, as assessed with both the self-report questionnaire and the proprioceptive drift (Bolognini et al., 2014; Burin et al., 2015; Llorens et al., 2017). This suggests a more flexible sense of body ownership in these patients.

Most trials exploring the use of VR for stroke have been focused on rehabilitation (Perez-Marcos et al., 2012), with some promising results for regaining of function (for a systematic review, see Laver et al., 2017). Our search did not find any studies of immersive VR interventions specifically for post-stroke pain. However, in a single case report, a 50-year-old woman with weakness, spasticity, sensory loss, and burning pain in the left upper limb underwent 10 sessions of conventional mirror therapy in 2 weeks, with significant pain relief post-treatment that was maintained at 1-year follow-up (Corbetta et al., 2018). The potential exists then for VR treatments that provide illusory movement of an embodied arm, though this has yet to be explored.

### Other central pain syndromes

The extent to which body image is altered in other central pain syndromes is relatively unexplored; however, it follows that since perceptual disorders are intrinsically linked to pain and sensorimotor deficits in many other neurological diseases (Moseley and Flor, 2012), they are likely to play a role.

Multiple sclerosis (MS) is a common autoimmune disease in which the myelin sheaths of the central nervous system are attacked and destroyed by the body’s immune system, causing significant sensory and motor deficits as well as pain. Pain is present in about 40% of cases, but only about 5% of this is neuropathic-type pain. MS pain is strongly correlated with fatigue, depression, and disability (Heitmann et al., 2020); however, any link between pain and perceptual deficits is not clear.

Our review did not find any published immersive VR interventions for MS. However, Nava et al. (2018) compared the rubber hand illusion in patients with relapsing-remitting MS and healthy controls and found that, while there was no difference in their reported subjective ownership of the rubber hand between groups, in the MS patients there was no proprioceptive drift towards the rubber hand. They speculate that MS affects primarily the self-location components of bodily self-consciousness while leaving unaltered the sense of body ownership, which requires additional top-down processes that are less affected by the myelin loss seen in MS. Perhaps VR interventions that focus on retraining the user’s proprioception may potentially be of benefit.

## Conclusion and Future Research

While showing promise, the use of virtual embodiment and body transformation illusions for neuropathic pain conditions requires higher-quality research, with large randomized clinical trials, before reaching any firm conclusions regarding clinical efficacy. Additionally, we would argue that any clinical trials should be pragmatic, reflecting real-world usage. The technological and cost revolution that has occurred in virtual reality over the last few years has made these powerful bodily illusion and embodiment tools readily available to the general public, facilitating their use at home or in outpatient clinics. However, its use in these settings is still very new, and there are many ethical and practical considerations that need to be explored (Donegan et al., 2020). In these early stages, the deep involvement and detailed feedback of end-users are essential when in the design phase of clinical trials.

The complex multi-systemic nature of neuropathic pain means that any single treatment used in isolation is highly unlikely to be effective. While treatments that aim to target and reverse distorted body perceptions associated with neuropathic pain have shown some therapeutic promise, they will not be effective for every neuropathic condition and every individual. A significant challenge will be whether we can easily identify patients that are more likely to respond to such treatments, since altered bodily perceptions can often be subtle and easily missed in clinical assessments, or patients may not volunteer such information for fear of being stigmatized. The use of validated patient reported outcome measures such as the Bath CRPS Body Perception Disturbance Scale (Lewis and McCabe, 2010) could be of help, and equivalent valid and reliable measures are required for other neuropathic pain conditions for both the initial identification of these disturbances as well as accurately monitoring their progress during treatment.

Additionally, much remains unknown and controversial regarding the physiological mechanisms of the effect of these treatments. The maladaptive cortical representations model, once widely accepted, has come under increasing scrutiny for example. More advanced neuroimaging techniques with better spatial and temporal resolution are now much more widely available and should be used in conjunction with non-invasive measures of neural function such as transcranial magnetic stimulation (TMS)/EMG, to try to clarify these underlying mechanisms.

Finally, little is known about the optimal dosage for bodily illusions. Whether repeated treatments prolong any analgesic effect is not clear, nor is the minimal nor optimal time for the length or number of treatment sessions known. Also, whether there is a cumulative benefit for combining bodily illusions with other brain-based treatments such as TMS should be explored. It is likely, given the heterogeneous nature of chronic pain, that the most clinically effective way forward will be a targeted and individualized multi-treatment approach, but whether this can be delivered cost-effectively remains to be seen. Even still, the fact that these treatments have the potential to help address a series of previously intractable and highly disabling pain conditions is hugely exciting.

## Author Contributions

All authors contributed to the bibliographic review and writing of the manuscript. All authors contributed to the article and approved the submitted version.

## Funding

This work was supported by the Commission for Universities and Research of the Department of Innovation, Universities, and Enterprise of the Generalitat de Catalunya -AGAUR- (IU16-011508) and co-financed by the European Union Regional Development Fund within the framework of the ERDF/FEDER Operational Program of Catalonia 2014–2020 with a grant of 50% of total eligible cost.

## Conflict of Interest

The authors declare that the research was conducted in the absence of any commercial or financial relationships that could be construed as a potential conflict of interest.

## Publisher’s Note

All claims expressed in this article are solely those of the authors and do not necessarily represent those of their affiliated organizations, or those of the publisher, the editors and the reviewers. Any product that may be evaluated in this article, or claim that may be made by its manufacturer, is not guaranteed or endorsed by the publisher.
